# Functionalization of Artwork Packaging Materials Utilizing Ag-Doped TiO_2_ and ZnO Nanoparticles

**DOI:** 10.3390/molecules29153712

**Published:** 2024-08-05

**Authors:** Tilde de Caro, Roberta Grazia Toro, Luminita Cassone, Francesca Irene Barbaccia, Camilla Zaratti, Irene Angela Colasanti, Mauro Francesco La Russa, Andrea Macchia

**Affiliations:** 1 CNR-ISMN, Istituto per lo Studio dei Materiali Nanostrutturati, Strada Provinciale 35 d n. 9, 00010 Rome, Italy; robertagrazia.toro@cnr.it; 2 Youth in Conservation of Cultural Heritage (YOCOCU APS), Via T. Tasso 108, 00185 Rome, Italy; luminitacassone.lc@gmail.com (L.C.); colasanti.1748988@studenti.uniroma1.it (I.A.C.); andrea.macchia@unical.it (A.M.); 3 Department of Technological Innovation Engineering, Digital Technologies for Industry 4.0, International Telematic University Uninettuno, Corso Vittorio Emanuele II 39, 00186 Rome, Italy; 4 Lab4Green, Via T. Tasso 108, 00185 Rome, Italy; zaratti.1920859@studenti.uniroma1.it; 5 Department of Biology, Ecology and Earth Sciences (DIBEST), University of Calabria, Via Pietro Bucci, Arcavacata, 87036 Rende, Italy; mlarussa@unical.it

**Keywords:** storage, packaging, smart, museum, nanomaterials, TiO_2_, ZnO, Ag doping

## Abstract

Most of the artworks stored in museums are often kept in inappropriate climatic and environmental conditions that facilitate the formation and growth of microorganisms, such as fungi, which are responsible for many types of biodegradation phenomena. To mitigate and prevent these deteriorative processes, functionalized packaging materials can be used for the storage and handling of artworks. The aim of this study was to develop a potential anti-biodeterioration coating suitable for packaging purposes. TiO_2_ and ZnO doped with different amounts of Ag (0.5 wt%, 1 wt%, and 3 wt%) were synthesized and dispersed in polyvinyl alcohol (PVA) and acrylic resin (Paraloid B72), then applied on different types of packaging materials (cellulose and the high-density spunbound polyethylene fiber Tyvek^®^, materials that are frequently used as packaging in museums). Analytical investigations (SEM/EDS, Raman, FTIR, and XRD) were employed to assess dispersion on the packaging material. Furthermore, resistance against biodeteriogens was assessed using *Cladosporium* sp., a bioluminometer, to define the biocidal efficacy.

## 1. Introduction

The preservation of cultural heritage artworks is of utmost importance, as these represent a testimony to the past and, more importantly, a legacy for future generations, a legacy that is up to us to maintain and preserve.

One of the problems that artworks face is fungal colonization and development. This is a typical issue for museums, particularly those housed in historic buildings with an unstable environment or extreme interior temperatures [1,2,3,4]. Air conditioning systems can control climatic factors, but this method is susceptible to fungi contamination and is difficult to operate in large areas due to high energy consumption [5]. As a result, some microorganisms and fungal species thrive in hot and humid conditions, within both historic buildings and modern climate-controlled structures [6,7]. These pathogens infiltrate museum spaces and storage through air currents or hitch a ride on visitors’ clothing, hair, bodies, or shoes [8]. Even a single contaminated item entering the museum environment can act as a carrier of contamination [9], leading to the surface degradation of artworks and impacting both coloration and structural integrity [10].

Additionally, artworks may be biologically attacked by microorganisms during their transportation from exhibition halls to storage or when they are loaned to other museum entities for temporary exhibitions. The most common microorganisms, specifically fungi, that attack and destroy artworks are *Aspergillus* spp., *Penicillium* spp., *Cladosporium* spp., *Mucor*, *Alternaria*, *Rhizopus* spp., and *Fusarium* [11,12,13].

Microbial and fungal metabolic processes oxidize, hydrolyze, and decompose organic artworks. Cellulose-based materials such as paper, photographs, fabrics, canvas paintings, and wood sculptures are susceptible to “foxing” and selective fungal infections on hemicellulose portions of the material [14,15,16,17]. However, microbes are not selective; they can attack inorganic materials, leading to structural problems and chromatic alterations [18,19,20]. Fungal activity, for example, alters porosity and surface compactness and can cause weight loss in stone structures [21,22].

Several scientific investigations have examined possible strategies to avoid the spread of microorganisms on artworks and in museum rooms and storage. Methods include chemical biocide attacks and physical mechanisms such as laser or microwave treatments to create unfavorable environmental conditions for their growth and proliferation [23,24,25,26,27,28]. Despite the different strategies employed to fight microorganisms, spore transmission by external vectors continues to be an important driver of contamination [29].

Therefore, safeguarding museum artworks against fungal attacks by using appropriate packaging could be essential to the long-term preservation of these art pieces [30,31,32]. Packaging materials should operate as a barrier to biocontamination, limiting the spread of spores within museum rooms and storage. The type of support and protection required for the item will be determined by the storage environment and the artwork itself; therefore, both should be considered.

Traditional packaging, while useful for protecting the material from stresses during transport and variations in irradiance and hygrometric conditions, may still allow microorganism spores to pass through, allowing them to proliferate on the artwork if the conditions are favorable [33,34].

To avoid contamination of works of art, the packaging utilized should, in addition to being appropriate for the type of art, be functionalized against microbes. Antimicrobial packaging technologies to prevent microbial growth have been widely studied and employed in food packaging. Several antimicrobial packaging technologies, involving different materials, have been investigated [35,36,37,38,39,40,41].

In food packaging, the formulation of an active protection system can provide additional protection compared to simple classical packing methods. Depending on their different applications, packaging can provide different functions, making it highly customizable [42,43]. For example, some innovative solutions to extend the shelf life of food by inhibiting or retarding microbial growth use the action of antimicrobial compounds incorporated within the packaging system [42].

Polymeric materials are commonly utilized in the production of this type of packaging. Creating multilayered packaging is the best way to protect food. However, this approach can be costly; an alternative is to incorporate compounds, such as nanoparticles, into the polymeric material to achieve a similar level of protection [44]. A tailored solution for the cultural heritage field still needs to be investigated [45].

Non-acid-free materials such as cellulose paper or Tyvek^®^ (a material produced by Du Pont (Wilmington, DE, USA) made of polyethylene fibers (PE) or rigid plastics such as polycarbonates (Makrolon^TM^) and acrylics (Perspex^TM^, Plexiglas^TM^)), as well as foams such as latex, are all examples of materials that should be avoided [31,46,47,48].

The aim of this study is to functionalize commonly used packaging materials for cultural heritage by using nanoparticles (NPs) that exhibit antimicrobial properties. Through the incorporation of NPs in two different mediums, the goal is to develop antifungal coatings that can be applied on packaging material to inhibit the growth of microorganisms.

Based on antibacterial, antifouling, and photocatalytic properties, TiO_2_ and ZnO NPs were chosen to increase the protective biocide efficacy of packaging materials [23,30,49,50]. Previous research on ZnO NPs has highlighted a correlation between reduced NP dimensions and increased concentration in inhibiting fungal growth [51]. TiO_2_ and ZnO were further investigated by incorporating Ag as a doping agent to enhance their effectiveness by increasing their adhesion on the chosen substrate [52,53].

Ag-doped TiO_2_ and ZnO NPs were synthesized using simple and environmentally friendly sol-gel methods, devoid of toxic and harmful solvents. To enhance adhesion between the substrates (a paper sheet and Tyvek^®^) and the coating, Ag-doped NPs were dispersed in two polymeric media consisting of polyvinyl alcohol (PVA) and acrylic resin (Paraloid B72).

Poly(vinyl alcohol) (PVA) and poly(methyl methacrylate) (PMMA) are the most commonly studied polymers for incorporating inorganic nanofillers. Nanomaterials are increasingly seen as superior alternatives to traditional additives for enhancing polymer properties.

The use of nanoparticles as polymer fillers aligns with the current strong interest in developing and applying new composite materials. Polymer nanocomposites effectively combine the unique size-dependent capabilities of nanoparticles with the desirable qualities of host polymers, such as versatility in design, long-term stability, and reprocessability. While the properties of nanocomposites generally reflect the combined attributes of their inorganic and organic components, synergistic interactions can sometimes lead to entirely new characteristics [54,55].

PVA is a non-toxic, water-soluble polymer widely utilized as a matrix material for synthesizing highly transparent nanocomposites due to its film-forming capability, biocompatibility, and cost efficiency. Its chemical formula is [CH_2_-CH (OH)]n, where n denotes the number of polymerizations. As reported by Huang et al. [56], the concentration of PVA is a critical factor because low concentrations generally lead to coatings with poor adhesion, while high concentrations cause PVA to become viscous and unsuitable for spraying.

Paraloid B72 was developed as a surface coating and offers various benefits, including strength and hardness without brittleness. Paraloid B72 is also characterized by a high glass transition temperature (Tg) and is less likely to experience cold flow [57]. It has been reported that by adding nano-TiO_2_ particles to high-density polyethylene and other materials, it is possible to obtain useful novel materials for packing applications [58].

There are several studies about the application of Paraloid B72 functionalized with NPs for the consolidation and protection of stone surfaces; however, there are very few studies focused on the packaging of artworks [59,60,61,62,63].

The biocidal efficacy depends both on the physicochemical properties of the particles and their interaction with the medium used for the coating formulation. The physicochemical properties of TiO_2_ and ZnO can vary depending on the material’s primary particle size, crystallinity, and formulation. Different properties have been found to cause different toxicological responses. The biocidal efficacy of both titanium dioxide and zinc oxide nanoparticles, as well as doped formulations, is closely related to their structure, which must be characterized for their application [64,65,66].

This type of multifunctional coating is not new to the cultural heritage sector. However, in this study, we have developed and applied coatings for the first time to enhance the packaging used in museums.

The structure and composition of the synthesized materials were investigated by using different analytical investigations, and biocide efficacy against fungi was defined using a bioluminometer. The experimental results show that the synthesized composite materials are able to improve the packaging used in museums to preserve cultural heritage.

## 2. Results and Discussion

### 2.1. Characterization of TiO_2_-Ag NPs

**Figure 1:**
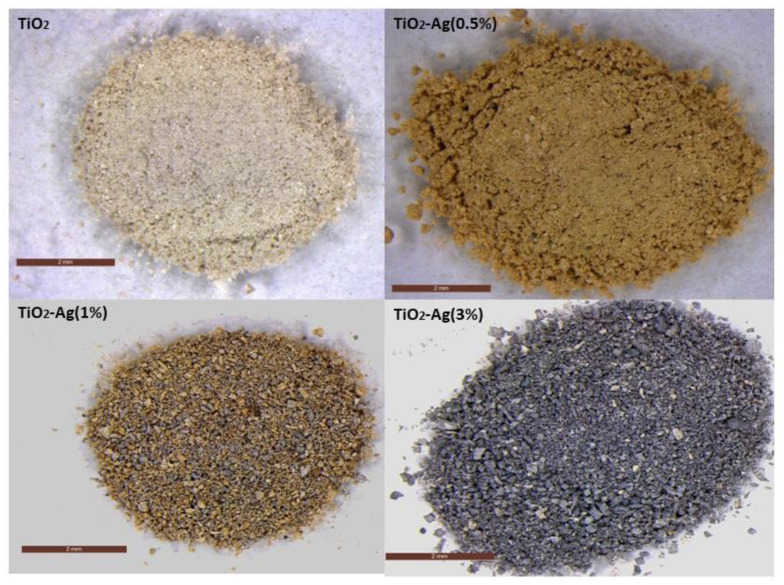
Stereomicroscope images of TiO_2_, TiO_2_-Ag (0.5%), TiO_2_-Ag (1%), and TiO_2_-Ag (3%).

TiO_2_-Ag NPs were synthesized using no harmful solvents and non-toxic reagents. The success of the adopted procedure was first approximately assessed by observing the morphological change in color of the obtained powders, ranging from white to brown, finally changing to deep grey upon increasing the amount of Ag doping (Figure 1).

**Figure 2:**
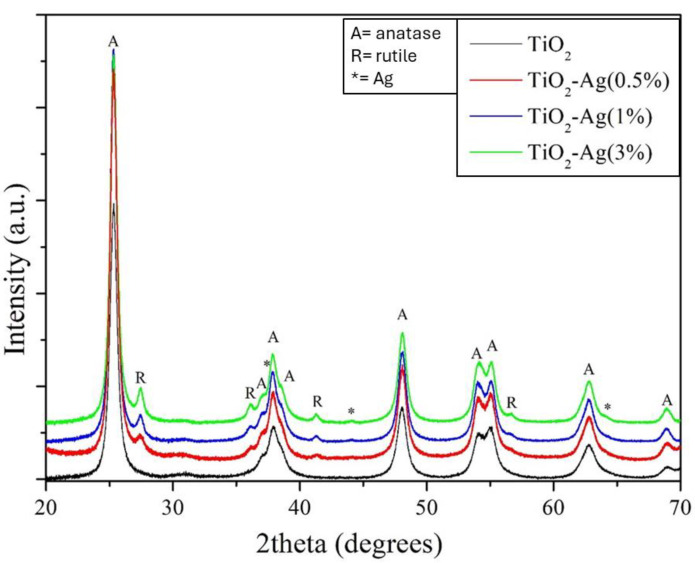
X-ray diffraction measurements of TiO_2_, TiO_2_-Ag (0.5%), TiO_2_-Ag (1%), and TiO_2_-Ag (3%). (A: anatase, R: rutile, * Ag).

Structural characterization of the obtained TiO_2_-Ag NPs was performed by X-ray diffraction measurements (Figure 2). Diffraction peaks positioned at 2θ of 25.31°, 36.92°, 37.82°, 38.55°, 48.05°, 53.98°, 55.09°, 62.79°, 68.89°, 70.56°, and 75.41° correspond to the pure TiO_2_ in the anatase phase (A), and were assigned to (101), (103), (004), (112), (200), (105), (211), (204), and (116) crystallographic planes (JCPDS card no. 21-1272) [67]. Moreover, the appearance of typical diffraction peaks (111), (200), and (220) positioned at 2θ of 38.10°, 44.39°, and 64.70 indicated the face-centered cubic metallic Ag crystal structure (JCPDS Card no. 04-0836) [68]. The characteristic XRD peak positions of anatase were scarcely affected by Ag doping, thus suggesting that Ag NPs did not enter the TiO_2_ crystal structure, causing distortion in the TiO_2_ crystal lattice, but were mainly on the surface of TiO_2_ NPs. Furthermore, XRD measurements also indicated the presence of rutile (R) as a secondary phase in all Ag-doped samples, characterized by diffraction peaks at 2θ of 23.43°, 36.05°, 41.25°, and 56.61°, which are related to (110), (101), (111), and (220) crystallographic planes (JCPDS card no. 21-1276) [69]. To determine the effect of doping with silver on the crystallite size of the nanoparticles, the mean crystallite size was calculated from the highest diffraction peak and full width at half maximum (FWHM) using the Debye–Scherrer equation (Equation (1)):

D (nm) = 0.9λ/βCosƟ (1)

where β is the FWHM (Full width at half maximum), λ is the X-ray wavelength of Cu-Ka (1.5406 A°), and Ɵ is the diffraction angle of the particles. The average crystallite sizes of pure and Ag-doped TiO_2_ NPs were calculated by the Scherrer equation using the full width at half maximum (FWHM) of the (101) diffraction peak. The corresponding values are reported in Table 1. Calculations suggested that TiO_2_ crystallite size varied in the range of 12–13 nm, with a slight reduction in size upon increasing the amount of Ag doping (Table 1).

**Figure 3:**
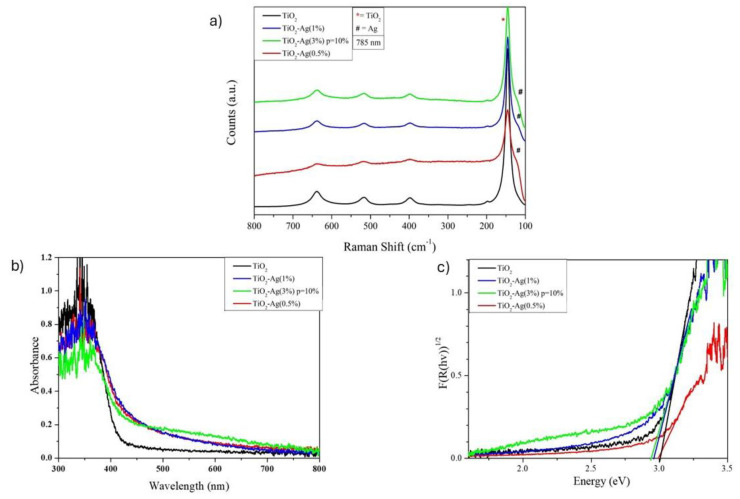
Spectra of TiO_2_ and TiO_2_-Ag (0.5%, 1%, 3%) acquired from different analyses, respectively. (**a**) Micro-Raman spectra; (**b**) UV–Vis spectra; (**c**) Kubelka–Munch.

Figure 3a shows the Raman spectra for different TiO_2_-Ag NPs. In the same picture, the Raman spectrum of undoped TiO_2_ NPs is shown for comparison. According to factor group analysis, TiO_2_ in the anatase phase has six Raman active modes (A1g + 2B1g + 3Eg) that are visible at 145 cm^−1^ (Eg), 197 cm^−1^ (Eg), 399 cm^−1^ (B1g), 513 cm^−1^ (A1g), 519 cm^−1^ (B1g), and 639 cm^−1^ (Eg) [70]. A smaller particle size affects the force constants and vibrational amplitudes of the nearest neighboring bonds, resulting in observed Raman shifts from 144 to 145 cm^−1^ [71].

Figure 3a highlights that the Raman spectra of all the synthesized TiO_2_-Ag and undoped TiO_2_ NPs have the characteristic Raman active modes of the anatase phase. In addition, the Raman spectra of doped samples report the presence of a peak at 122 cm^−1^, characteristic of Ag^0^, which is likely to be formed during thermal annealing in air [72]. Therefore, the Raman spectra of TiO_2_ and TiO_2_-Ag did not exhibit any discernible differences in crystalline structure.

The optical absorption of Ag-doped and undoped TiO_2_ NPs was measured with diffuse reflectance UV/Vis absorption spectroscopy and compared in Figure 3b. As can be seen, for the undoped TiO_2_ NPs (the black curve in Figure 3b), the absorption spectrum showed typical semiconductor absorption properties, with strong absorption in the ultraviolet region and weaker absorption in the visible light region. Compared to pure TiO_2_, the absorption intensity in the visible region was much higher after Ag doping, which indicates that the Ag doping process enhances the absorption efficiency of the TiO_2_ photocatalyst in visible light. Furthermore, the absorption spectra showed significant absorption in the visible region proportional to Ag doping, and absorption potential became stronger upon increasing the Ag doping concentration. In addition, the absorption edges of the Ag-doped TiO_2_ NPs were significantly red-shifted compared to the undoped TiO_2_. This behavior, already reported in previous studies [73], was attributed either to the surface plasmon absorption of Ag nanoparticles or enhanced photon path length in the surface region of TiO_2_ NPs. The optical bandgap energies (Eg) of the synthesized samples were calculated according to the K–M equation and the values are reported in Table 1. Eg value was affected by Ag doping and its value started to decrease upon increasing doping concentration (Figure 3c), reaching the value of 2.88 eV for the sample with 3 wt% of Ag doping.

### 2.2. Characterization of ZnO-Ag NPs

**Figure 4:**
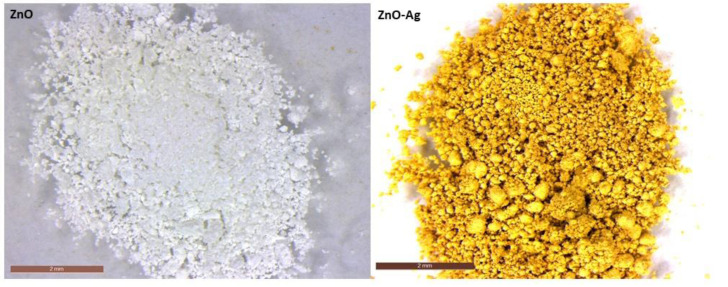
Stereomicroscope images of ZnO (**left**) and ZnO-Ag NPs (**right**).

In the case of Ag-doped ZnO NPs, the color of the synthesized powders changed with the dopant, varying from white for the undoped ZnO to gold-ochre for Ag-doped samples (Figure 4).

**Figure 5:**
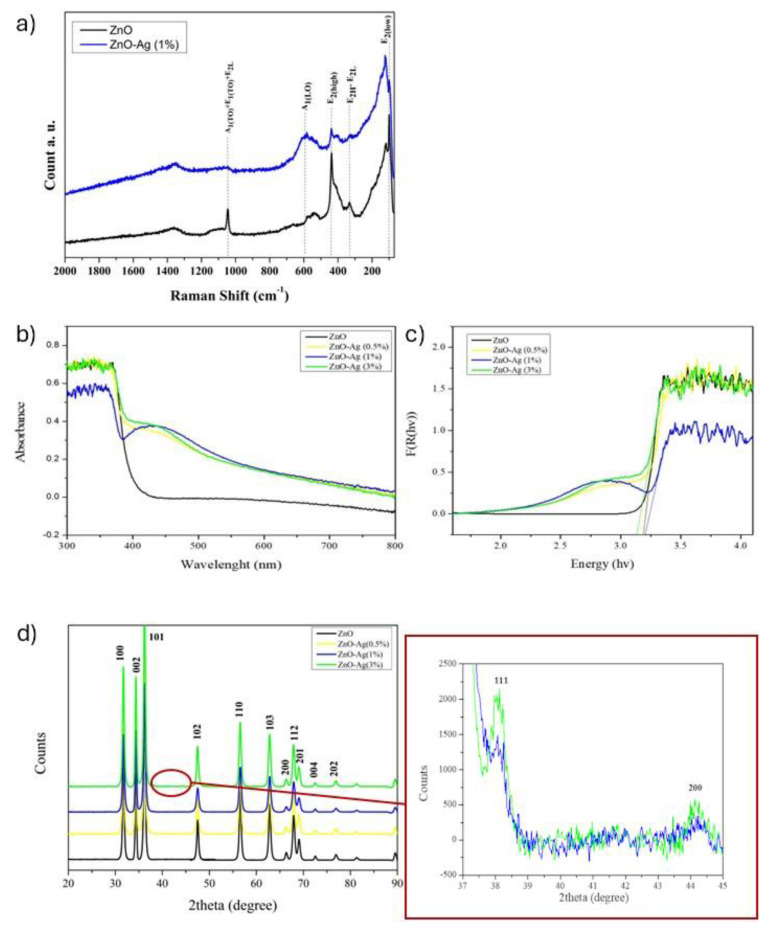
Spectra of ZnO and doped ZnO with Ag, respectively. (**a**) Micro-Raman spectra; (**b**) UV–VIS spectra recorded by diffuse reflectance spectroscopy; (**c**) Kubelka–Munch spectra; (**d**) XRD spectra.

Structural characterization carried out by X-ray Diffraction experiments confirmed the formation of ZnO in all the analyzed samples. XRD patterns (Figure 5d) showed the presence of peaks at 31.75°, 34.41°, 36.25°, 47.54°, 56.58°, 62.85°, 66.39°, 67.92°, and 69.06°, which can be related to the (100), (002), (101), (102), (110), (103), (200), (112), and (201) reflections of the hexagonal-type ZnO structure (JCPDS Card no. 043-0002) [74]. Furthermore, in the XRD pattern of Ag-doped ZnO with a nominal content of Ag equal to 1% and 3 wt%, two other peaks were clearly visible in addition to the ZnO peaks. These peaks at 2Ɵ values of 38.10° and 44.38° can be attributed to the (111) and (200) reflections of the cubic Ag structure. The reflections characteristic of Ag were not detected in the 0.5% ZnO-Ag, probably due to the low concentration of Ag dopant. As a dopant, Ag can exist as a substituent for Zn^2+^ in the ZnO lattice or as an intermediate space atom [75]. As a substitute for Zn^2+^, the Ag dopant should cause a peak shift in the corresponding XRD. Furthermore, none of the doped ZnO NP XRD patterns showed any significant shifts in peak positions. As suggested by Kareem et al. [75], Ag particles are likely to segregate at the grain boundaries of ZnO crystallites rather than going to the lattice of ZnO.

The effect of doping on the crystallite size of the nanoparticles was evaluated by using the Debye–Scherrer equation. The average sizes of undoped ZnO, 0.5% ZnO-Ag, 1% ZnO-Ag, and 3% ZnO-Ag are reported in Table 2. As can be seen, Ag doping causes a sensible decrease in crystallite size [76].

The structure of ZnO-Ag powders was further investigated by µ-Raman spectroscopy. Raman spectra are reported in Figure 5a: the analysis defined shifts in position and intensity between pure and doped ZnO [77,78,79].

According to factor group analysis, the active Raman modes of the ZnO phase are visible at 100 cm^−1^ (E_2L_), 331 cm^−1^ (E_2h_-E_2L_), 437 cm^−1^ (E_2H_), and 580 cm^−1^ (A1_LO_) [80].

The intensity of the peak at 437 cm^−1^ decreases with the addition of Ag because the ZnO structure becomes more defective; nonetheless, the peak at 578 cm^−1^ is marked by an increase in intensity, shift, and the broadening of peaks due to the reduction in crystallite size.

The reduction in crystallite size exerts an influence on the force constants and vibrational amplitudes of adjacent bonds. This, in turn, induces the red-shift and broadening of peaks in the resulting spectra of doped ZnO-Ag.

Diffuse reflectance spectroscopy (UV–Vis DRS) was employed to examine the light absorption properties of undoped and Ag-doped ZnO NPs. For all the samples, absorption increased sharply in the ultraviolet region due to the occurrence of band gap absorption. The UV–Vis spectra exhibited a shift in band edge, depending on the variation in Ag content. In the case of Ag-doped samples, the absorption of visible light is dominated by surface plasmon resonance (SPR) resulting from Ag nanoparticles.

Using the KM equation, the optical band gap values (Eg) were calculated, and they are presented in Table 2. Band gap energy is reduced with increased Ag doping for all nanoparticles. The observed variation in band gap values of ZnO (Figure 5c) with Ag doping may be attributed to the creation of oxygen vacancies, which introduce new intermediate energy levels between the valence (VB) and the conduction (CB) bands, responsible for reducing the final bandgap [81].

### 2.3. Application of Nanoparticles on Packaging

First, 1 wt% Ag-doped TiO_2_ and 1 wt% Ag-doped ZnO NPs were dispersed in PVA and in a commercial acrylic resin, namely Paraloid B72. Then, they were applied onto paper and polyethylene foils using an airbrush from Aerograph. For comparison, undoped TiO_2_ and ZnO were also used.

**Figure 6:**
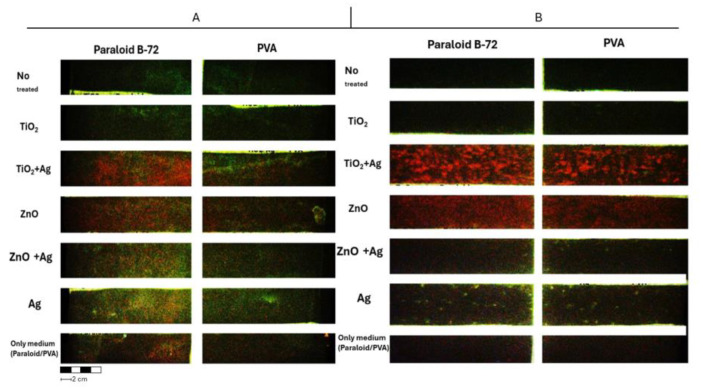
NP UV analysis images of the paper sample (**A**) and the Tyvek^®^ sample (**B**).

Figure 6 shows the dispersion of nanoparticles (NPs) on polyethylene (PE) and paper substrates under UV light observation. Under UV light, all formulations are characterized by a dark orange fluorescence, except for TiO_2_, where fluorescence is absent, probably due to its high UV absorption ability.

**Figure 7:**
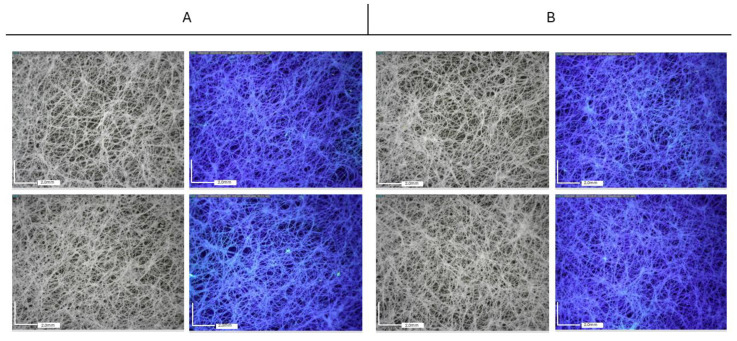
Paper packaging. Optical microscopy images (50×) acquired using visible light (**left**) and UV light (**right**). (**A**) Paraloid B72 medium (**top**) and PVA (**bottom**) + TiO_2_ NPs; (**B**) Paraloid B72 medium (**top**) and PVA (**bottom**) + TiO_2_-Ag NPs.

**Figure 8:**
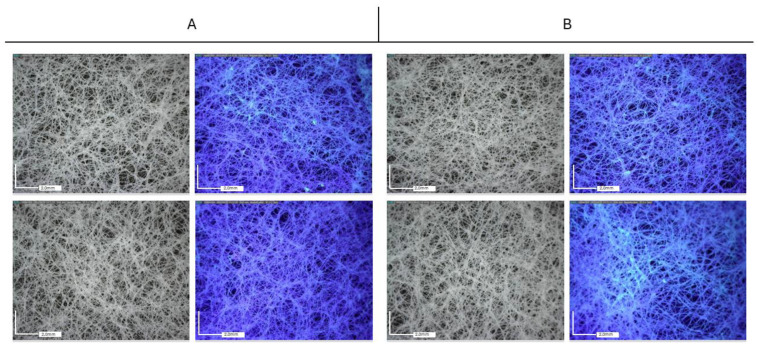
Paper Packaging. Optical microscopy images (50×) acquired using visible light (**left**) and UV light (**right**). (**A**) Paraloid B72 medium (**top**) and PVA (**bottom**) + ZnO NPs; (**B**) Paraloid B72 medium (**top**) and PVA (**bottom**) + ZnO-Ag NPs.

Figure 7 and Figure 8 report the observations collected by optical microscopy on treated paper foils. A whitish appearance was evident in the films containing both TiO_2_ and TiO_2_-Ag NPs. Additionally, a different distribution of particles was observed depending on the type of resin. In both cases, Paraloid B72-based formulations resulted in a better distribution of nanoparticles in comparison to PVA, which, in turn, showed a higher tendency for particle aggregation.

**Figure 9:**
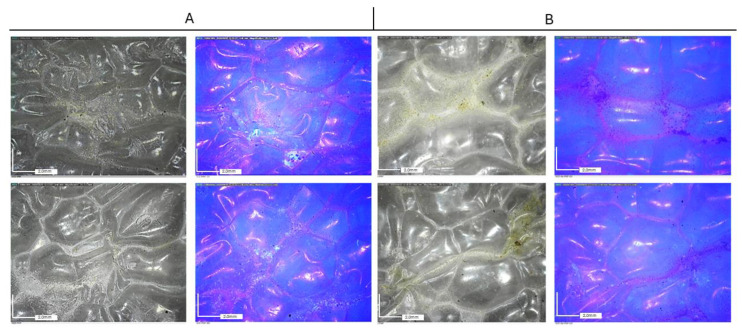
Tyvek^®^ packaging. Optical microscopy images (50×) acquired under visible light (**left**) and UV light (**right**). (**A**) Paraloid B72 medium (**top**) and PVA (**bottom**) + TIO_2_ NPs; (**B**) Paraloid B72 medium (**top**) and PVA (**bottom**) + TIO_2_-Ag NPs.

**Figure 10:**
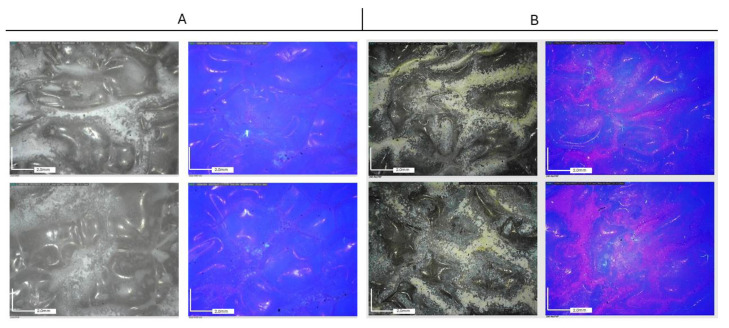
Tyvek^®^ packaging. Optical microscopy images (50×) acquired under visible light (**left**) and UV light (**right**). (**A**) Paraloid B72 medium (**top**) and PVA (**bottom**) + ZnO NPs; (**B**) Paraloid B72 medium (**top**) and PVA (**bottom**) + ZnO-Ag NPs.

Optical microscope observations collected on Tyvek^®^ (Figure 9 and Figure 10) have highlighted the non-uniform distribution of the NPs, which tend to aggregate mainly around the wrinkles of the substrate, adhering to the surface irregularities of the substrate and consequently aggregating in limited areas.

In both cases, a better distribution of nanoparticles is achieved when Paraloid B72 is used as the dispersant medium.

Table 3 shows the total chromatic variation calculated as the color difference between the substrates with coatings compared to the coating treatment with nanoparticles.

Comparing the Tyvek^®^ substrate to paper, only minor and noticeable chromatic variations (fewe than five) were found.

The coatings of ZnO and TiO_2_ induce a chromatic variation that is imperceptible to the naked eye compared to the coatings with doped Ag particles. The range of values between 2.58 and 5.64 indicates that all nanoparticles are present on all treated surfaces.

**Figure 11:**
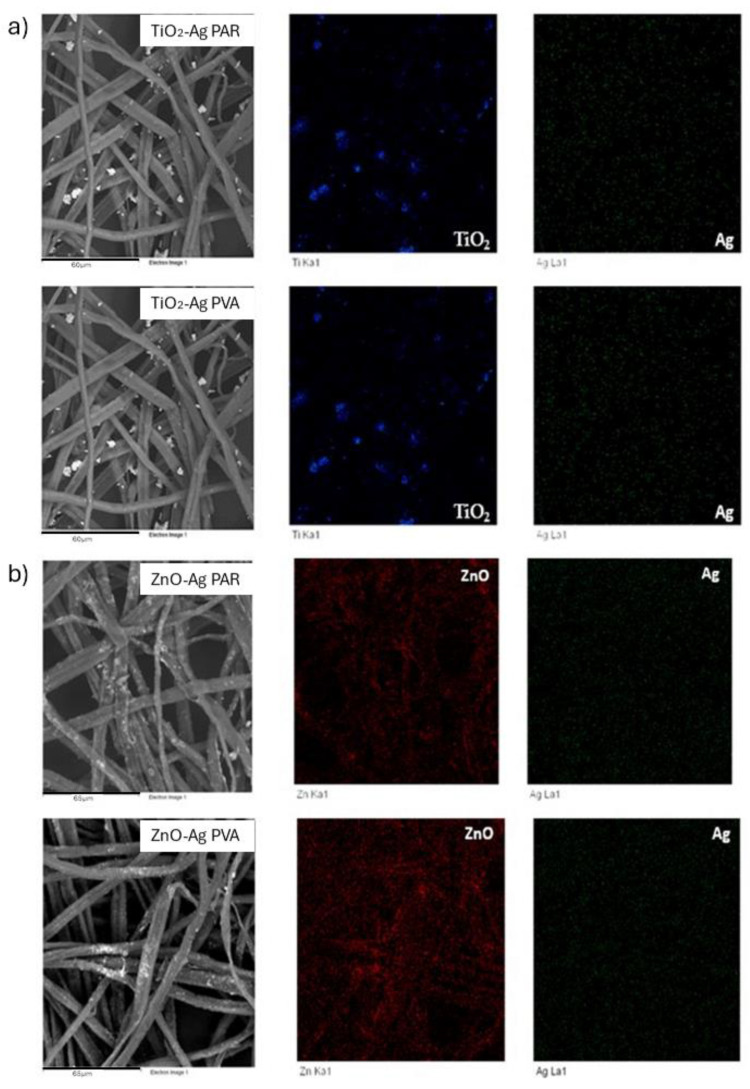
SEM analysis of NP distribution on packaging paper; (**a**) spatial distribution of TiO_2_-Ag NPs in Paraloid B72 (TiO_2_-Ag PAR) (**top**) and PVA (TiO_2_-Ag PVA) (**bottom**); (**b**) spatial distribution of ZnO-Ag NPs in Paraloid B72 (ZnO-Ag PAR) (**top**) and PVA (ZnO-Ag PVA) (**bottom**).

**Figure 12:**
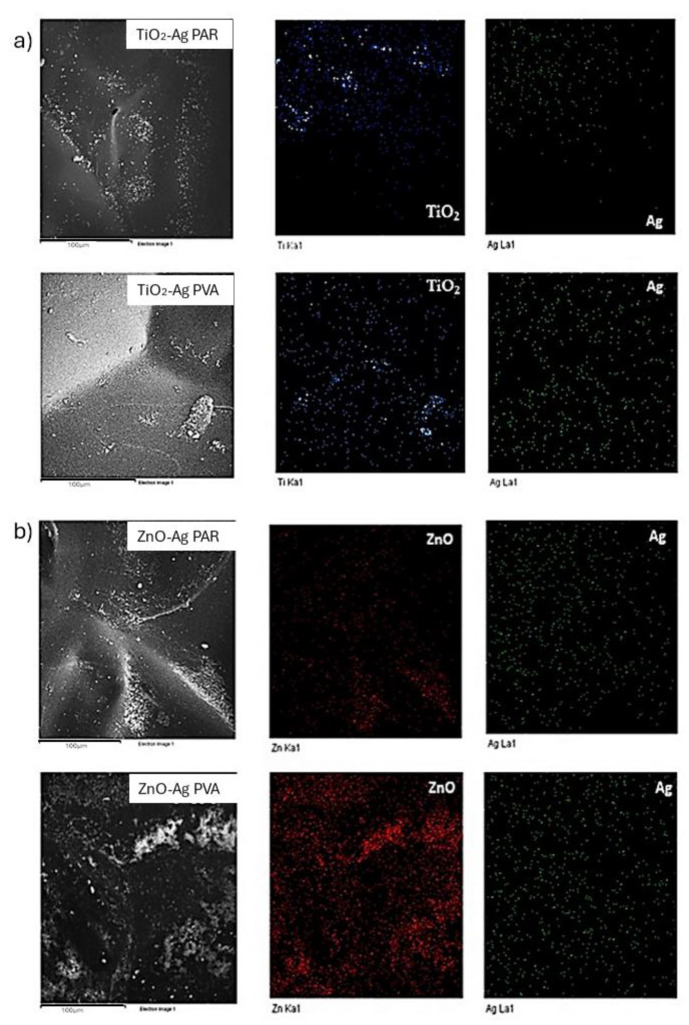
SEM analysis of NP distribution on PE; (**a**) spatial distribution of TiO_2_-Ag NPs in Paraloid B72 (TiO_2_-Ag PAR) (**top**) and PVA (TiO_2_-Ag PVA) (**bottom**); (**b**) spatial distribution of ZnO-Ag NPs in Paraloid B72 (ZnO-PAR) (**top**) and PVA (ZnO-Ag PVA) (**bottom**).

The surface morphology of the treated samples was analyzed by scanning electron microscopy (SEM). Figure 11 displays images of the paper sample and Figure 12 shows images of Tyvek^®^ surfaces coated with doped ZnO-Ag and TiO_2_-Ag NPs. SEM investigation confirmed that Zn-Ag nanoparticles dispersed in both PVA and Paraloid were more evenly distributed than TiO_2_-Ag-based formulations, which instead showed a sensible tendency to aggregate.

µ-Raman spectroscopy results confirmed the successful application of TiO_2_-Ag and ZnO-Ag NPs on both Tyvek^®^ and paper samples: in all Raman spectra, it is possible to identify the characteristic peak at 144 cm^−1^, 437 cm^−1^, 2886 cm^−1^, and 2892 cm^−1^, typical of TiO_2_, ZnO, PVA, and Paraloid B72, respectively (Figure S1, Table S1).

## 3. Microbiological Analysis

**Figure 13:**
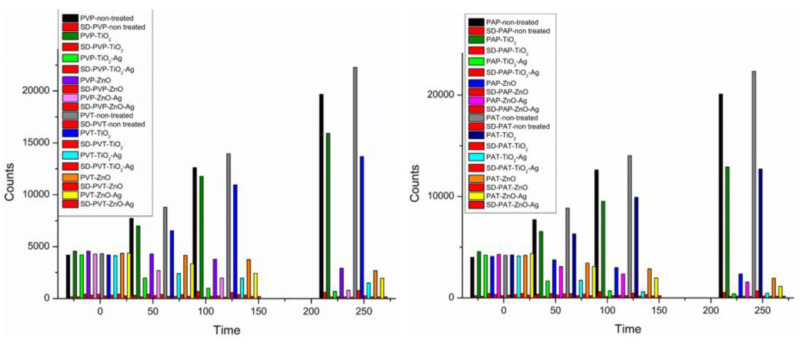
ATP values for several packing materials treated with the different experimental coatings.

The results from the fungal attack (Figure 13) define a significant proliferation of microbial species on untreated surfaces. The ATP (adenosine triphosphate, a marker for surface contamination of biological origin) data obtained indicate substantial differences in microbial vitality reduction between the two substrates considered in the experiment, with greater efficacy observed for coatings applied on paper compared to Tyvek^®^. This outcome is evident when employing PVA as a medium. The most notable variances were observed in the use of the two media; samples treated with Paraloid B72, as opposed to those treated with PVA, demonstrate a greater inhibition of biological growth for an equivalent amount of added nanoparticles. Regarding nanoparticles, the film characterized by the presence of TiO_2_ does not inhibit growth, although a slight decrease in ATP is still observed. The most favorable outcomes are achieved by functionalizing the media with TiO_2_ and ZnO doped with silver. Even the use of simple zinc oxide allows for a reduction in biological growth, albeit with less efficacy compared to the same particle doped with Ag. In all highlighted cases, complete inhibition of microbiological growth is not achieved. Silver-doped zinc oxide nanoparticles exhibit superior antibacterial activity compared to pure zinc oxide nanoparticles, as reported in the literature [82].

The incorporation of the metal dopant enhances the photocatalyst’s efficiency by reducing band gap energy and decreasing the rate of electron–hole recombination. The inefficacy of pure TiO_2_ as a biocide is due to its absorption in the ultraviolet region, where its photocatalytic activity is very high. By doping with metals, the absorption region of TiO_2_ is extended to the visible part of radiation. Doping facilitates the formation of hydroxyl and superoxide radicals, which are strong oxidizers; these radicals are the basis of the biocidal properties observed in doped TiO_2_ compared to undoped TiO_2_ [83]. Incubation of the samples was carried out under visible light illumination. The ineffectiveness of undoped TiO_2_ compared to undoped ZnO is attributed to the absence of UV radiation in the conducted tests.

## 4. Materials and Methods

### 4.1. Reagents

Titanium (IV) Butoxide (reagent grade, 97% Sigma-Aldrich, Milan, Italy), Zinc Acetate dihydrate (ACS reagent, ≥98% Sigma Aldrich), AgNO_3_ (ACS reagent, ≥99.0% Sigma-Aldrich), Citric Acid (ACS reagent, ≥99.5% Sigma-Aldrich), and Ethanol (99.8% Sigma-Aldrich) were used without any further purification. Double distilled water was used in all experiments. Cellulose paper packaging was provided by IMAR Italia and Tyvek^®^ (polyethylene) was provided by DuPont (Wilmington, DE, USA).

### 4.2. Synthesis of Nanoparticles

#### 4.2.1. Synthesis of TiO_2_-Ag Nanoparticles

TiO_2_-Ag NPs with different amounts of Ag doping (0.5, 1, and 3 wt%) were synthesized using a simple and green sol-gel approach in accordance with the procedure reported by Souza et al. [84], which was opportunely modified. First, 6.8 mL of titanium butoxide [Ti(OBu)_4_] was mixed with 2.9 mL of ethanol in a beaker and stirred vigorously for 30 min (solution A). In another beaker, 4.80 g of citric acid and 39.6 mL of deionized water (solution B) were mixed and a pH of ~2.0 was measured. Solution A was added dropwise to solution B under stirring, and the resulting solution was kept at 80 °C under reflux and constant stirring for 2 h to ensure the complete hydrolysis of the titanium precursor. The resulting clear and colorless hydrosol solution was allowed to cool down to room temperature. A certain volume of NH_4_OH 1 M was slowly added to TiO_2_ hydrosol until a pH of 5 was measured. After that, different amounts of AgNO_3_ were added, and the obtained hydrosols were sonicated at 80 W for 1 h at a temperature of 60 °C. Brownish Ag-doped TiO_2_ powders were obtained by solvent evaporation at 100 °C for 24 h. Crystalline Ag-doped TiO_2_ NPs were finally synthesized after two-step thermal annealing at 400 °C for 1 h (heating ramp: 1 °C/min) and then at 500 °C for 2 h (heating ramp 1 °C/min) (Figure 1). TiO_2_ NPs, except for AgNO_3_, were obtained following the same procedure.

#### 4.2.2. Synthesis of ZnO-Ag Nanoparticles

Ag-doped ZnO NPs were obtained by the following procedure: in a typical reaction process, 0.02 mol of Zn(NO_3_)_2_·6H_2_O was dissolved in 30 mL of distilled water (DW) at room temperature and under continuous stirring. Different amounts of 0.1 M AgNO_3_ solution were added to the clear solution to obtain a nominal dopant concentration of 0.5, 1, and 3 wt%, followed by the addition of a few drops of 1 M NaOH solution until a pH of 11 was reached. The obtained solution was first kept under agitation for 30 min at room temperature and then heated at 60 °C under reflux for 6h. The white precipitate was cooled down to room temperature and then was collected by centrifugation at 8000 rpm for 10 min and washed three times with water and ethanol. Finally, the powders were dried at 70 °C in a furnace for 12 h. No further thermal treatment was performed (Figure 4). Undoped ZnO NPs were obtained by the same procedure without the addition of AgNO_3_.

### 4.3. Preparation of TiO_2_-Ag/PVA and ZnO-Ag/PVA Formulations

**Figure 14:**
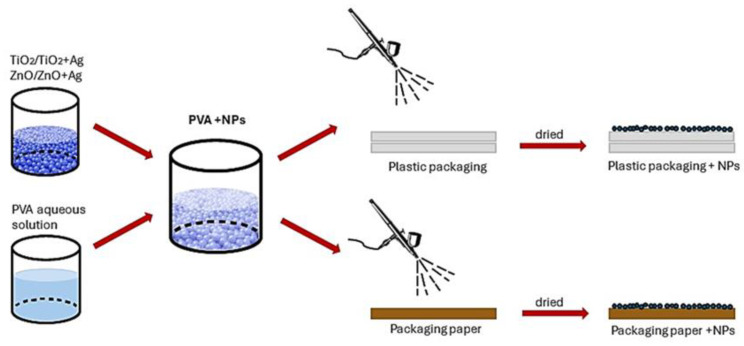
Procedure of TiO_2_/TiO_2_+Ag and ZnO/ZnO+Ag NP application on packaging materials.

The application of coating formulations is very simple (Figure 14). After NP synthesis, they were added to a PVA water solution to form an isotropic % by ultrasonication for 1 h. Then, the dispersion was sprayed onto the paper or PE surface and dried at room temperature.

For the preparation of the antimicrobial formulations, only TiO_2_-Ag and ZnO-Ag NPs with a nominal content of Ag equal to 1 wt% were used. In addition, undoped TiO_2_ and ZnO NPs were used as references. A fixed amount of nanoparticles (0.1 g) was dispersed in distilled water (10 mL) and ultrasonicated for 30 min at a power of 80 W in order to obtain a better dispersion of the nanoparticles in water. At this stage, a water solution of PVA (1 wt%) was added to the dispersion of nanoparticles to reach a final volume of 50 mL. The final solution was further stirred for 1 h at room temperature. Finally, after it was homogeneously stirred, the solution was sprayed on the surface of the paper and Tyvek^®^ by an aerographer.

To gain insight into the effects of PVA, two other formulations were set up using different concentrations of the PVA solution, namely 5 wt% and 10 wt%. Finally, the sprayed surfaces were allowed to dry at room temperature for 24 h.

The same procedure was followed for the preparation of other similar formulations in which the commercial acrylic resin Paraloid B72 was used as a substitution for PVA.

### 4.4. Methods

The optical properties of the NPs were defined using stereomicroscope analysis, performed with a Leica M125C stereomicroscope equipped with a DMC4500 USB digital camera (Leica, Wetzlar, Germany) and using a Jasco V630 UV–VIS spectrophotometer (Jasco, Tokyo, Japan) with a double ray, characterized by a silicon photodiode detector equipped with two sources of radiation. One of these is a deuterium lamp (190–350 nm) and the other is a halogen lamp (330–110 nm). To analyze the structure and crystallinity phases of the NPs, XRD analysis was carried out by means of a BrukerD8 advance theta-theta diffractometer (Bruker, Ettlingen, Germany) with a one millimeter point collimator with x, y, z adjustment, by laser and 3D detector.

The film formed by the coating was analyzed by UV fluorescence using a full spectrum NX500 28.2 MP BSI CMOS camera (Samsung, Suwon-si, Republic of Korea) with a Madetec UV lamp (Tec Lighting, Brea, CA, USA), at a wavelength of 365 nm.

To observe the distribution of NPs on the surface, digital microscope analysis was performed with Dino-lite AM4113t-FVW (Dino-Lite Digital Microscope, Bangkok, Thailand) in VIS and UV light (395 nm).

Colorimetric analysis was performed with a 3Nh TS7700 portable spectrophotometric colorimeter (3NH, Guangzhou, China). Spectrocolometry applied to the coatings was used to determine whether the treatment induced any color changes on the surface, highlighting the presence or absence of the treatment itself.

For the analysis of coatings and nanomaterials, micro-Raman spectroscopy was chosen because it is an accurate and noninvasive technique. The Raman spectra were obtained using a Renishaw RM 2000 Raman microscope (Renishaw, Hongkong, China) conjugated with a Leica optical microscope and equipped with a silicone CCD detector. The laser radiation was focused with the 50× and 100× lenses. The excitation source was a 785 nm diode laser. The actual power at the sample was 1.58 mW. The diffraction grating was 1800 L/mm. The accumulation time was 10 s with 10 repetitions. Two Edge filters blocked the Rayleigh-scattered light below 100 cm^−1^. For this reason, the study of ultra-low wavenumber Raman spectra in the region of <100 cm^−1^ is overlooked. To investigate the superficial morphology of the samples, an SEM TESCAN instrument was used, equipped with an X INCA 300 microprobe and a four-sensor retro diffuse electron detector (BSE) (TESCAN, Brno, Czechia). The beam acceleration voltage was set between 20 KeV and 30 Kev, which is sufficient to exceed the critical electron energy of the X-ray emission (quantitative resolution limit of 0.2% by weight).

To assess the stability to biological exposure of the tested formulations, the different coatings applied on paper and Tyvek^®^ were contaminated with pathogenic agents from *Cladosporium* sp., a widely distributed species in indoor environments [28,29] that belongs to the family of Dematiaceae. The samples were incubated 3 times at 60 °C for 240 min. Incubation of the samples was carried out under visible light illumination to simulate museum lighting conditions. The stability to biological attack of several coatings was defined by ATP measurements using a Lumitester PD 30 (Kikkoman, Chiba, Japan).

## 5. Conclusions

Within museums, not only are there exhibition sections for public viewing but there are also storage areas where artworks that do not find space in the exhibition areas are housed.

The biological degradation of materials is a critical concern in preserving cultural heritage, and the development of innovative nanoparticle-based packaging represents a promising solution to mitigate antifungal attacks on stored artworks.

This study has demonstrated the potential use of nanoparticulate biocides for functionalizing materials utilized in the packaging of artworks. For this purpose, Ag-doped ZnO and Ag-doped TiO_2_ NPs were synthesized and dispersed in two different polymeric mediums, namely PVA and Paraloid. This doping ensures enhanced biocidal efficacy of the particles, albeit with a chromatic variation nearly imperceptible to the human eye. SEM/EDS analysis highlighted the presence of nanoparticles on the surface of paper and Tyvek^®^, with a better homogeneous distribution of ZnO-Ag NPs than TiO_2_-Ag NPs. Ag-doped TiO_2_ exhibited the highest biocidal efficacy, attributed to its visible light-induced photocatalytic activity resulting from the doping process. Conversely, undoped TiO_2_ demonstrated no biocidal efficacy due to the absence of UV light-induced photocatalytic activation. Both doped and undoped ZnO nanoparticles exhibited good biocidal efficacy, although complete inhibition of microbial vitality (ATP) was not achieved in all cases.

This study contributes to the ongoing efforts to develop effective strategies for preserving cultural heritage materials during transport and storage, addressing the pressing issue of biological degradation.

## Figures and Tables

**Table 1:** TiO_2_ crystallite size (D), calculated by the Debye–Scherrer equation, and optical bandgap energies (Eg), calculated by the K–M equation.

Nanoparticles	TiO_2_			
	Undoped	0.5% Ag	1% Ag	3% Ag
D (nm)	12.33	13.09	12.89	12.15
Eg (eV)	3.00	2.99	2.92	2.88

**Table 2:** ZnO crystallite size (D), calculated by the Debye–Scherrer equation, and optical bandgap energies (Eg) calculated by the K–M equation.

Nanoparticles	ZnO			
	Undoped	0.5% Ag	1.0% Ag	3% Ag
D (nm)	22.96	20.54	18.96	21.37
Eg (eV)	3.21	3.19	3.21	3.14

**Table 3:** ΔE between the substrates with coatings compared to the coating treatment with nanoparticles. SD < 0.5.

Coating	Particles	Paper	Tyvek^®^
Paraloid B72	TiO_2_	2.58	3.91
	TiO_2_-Ag	3.44	5.60
	ZnO	4.73	4.01
	ZnO-Ag	3.75	6.61
PVA	TiO_2_	3.23	5.39
	TiO_2_-Ag	3.55	5.94
	ZnO	2.81	4.72
	ZnO-Ag	3.37	5.64

## Data Availability

The original contributions presented in the study are included in the article/Supplementary Material.

## Supplementary Materials

The following supporting information can be downloaded at: https://www.mdpi.com/article/10.3390/molecules29153712/s1. Figure S1. Micro-Raman spectra: (a) paper after TiO_2_ NPs and adhesives application; (b) paper after ZnO NPs and adhesives application; (c) Tyvek^®^ after TiO_2_ NPs and adhesives application; (d) Tyvek^®^ after ZnO NPs and adhesives application; were PAP is refer to Paraloid on paper, PAT is Paraloid on Tyvek^®^, PVP is PVA on paper and PVT is PVA on Tyvek^®^. Table S1. Raman peaks of packaging material studied. Refs. [84,85,86,87,88,89,90,91,92,93,94] are cited in the Supplementary Materials.
