# Supplementary material for: Functionalization of Artwork Packaging Materials Utilizing Ag-Doped TiO2 and ZnO Nanoparticles
Source: Molecules. 2024 Aug 5;29(15):3712. doi: 10.3390/molecules29153712 (PMC11314615; doi:10.3390/molecules29153712)
Supplement: Supplementary file 1 [file molecules-29-03712-s001.zip › molecules-3077873-supplementary.pdf]

Functionalization of Artwork Packaging Materials Utilizing Ag-Doped TiO_2 and ZnO Nanoparticles

Tilde de Caro [1,*,†], Roberta Grazia Toro [1,†], Luminita Cassone [2], Francesca Irene Barbaccia [2,3], Camilla Zaratti [4], Irene Angela Colasanti [2], Mauro Francesco La Russa [5] and Andrea Macchia [2,4]

1 CNR-ISMN, Istituto per lo Studio dei Materiali Nanostrutturati, Strada Provinciale 35 d n. 9, 00010 Rome, Italy; robertagrazia.toro@cnr.it

2 Youth in Conservation of Cultural Heritage (YOCOCU APS), Via T. Tasso 108, 00185 Rome, Italy; luminitacassone.lc@gmail.com (L.C.); colasanti.1748988@studenti.uniroma1.it (I.A.C.); andrea.macchia@unical.it (A.M.)

3 Department of Technological Innovation Engineering, Digital Technologies for Industry 4.0, International Telematic University Uninettuno, Corso Vittorio Emanuele II 39, 00186 Rome, Italy

4 Lab4Green, Via T. Tasso 108, 00185 Rome, Italy; zaratti.1920859@studenti.uniroma1.it

5 Department of Biology, Ecology and Earth Sciences (DIBEST), University of Calabria, Via Pietro Bucci, Arcavacata, 87036 Rende, Italy; mlarussa@unical.it

* Correspondence: tilde.decaro@cnr.it

† These authors contributed equally to this work.

(a) (b)

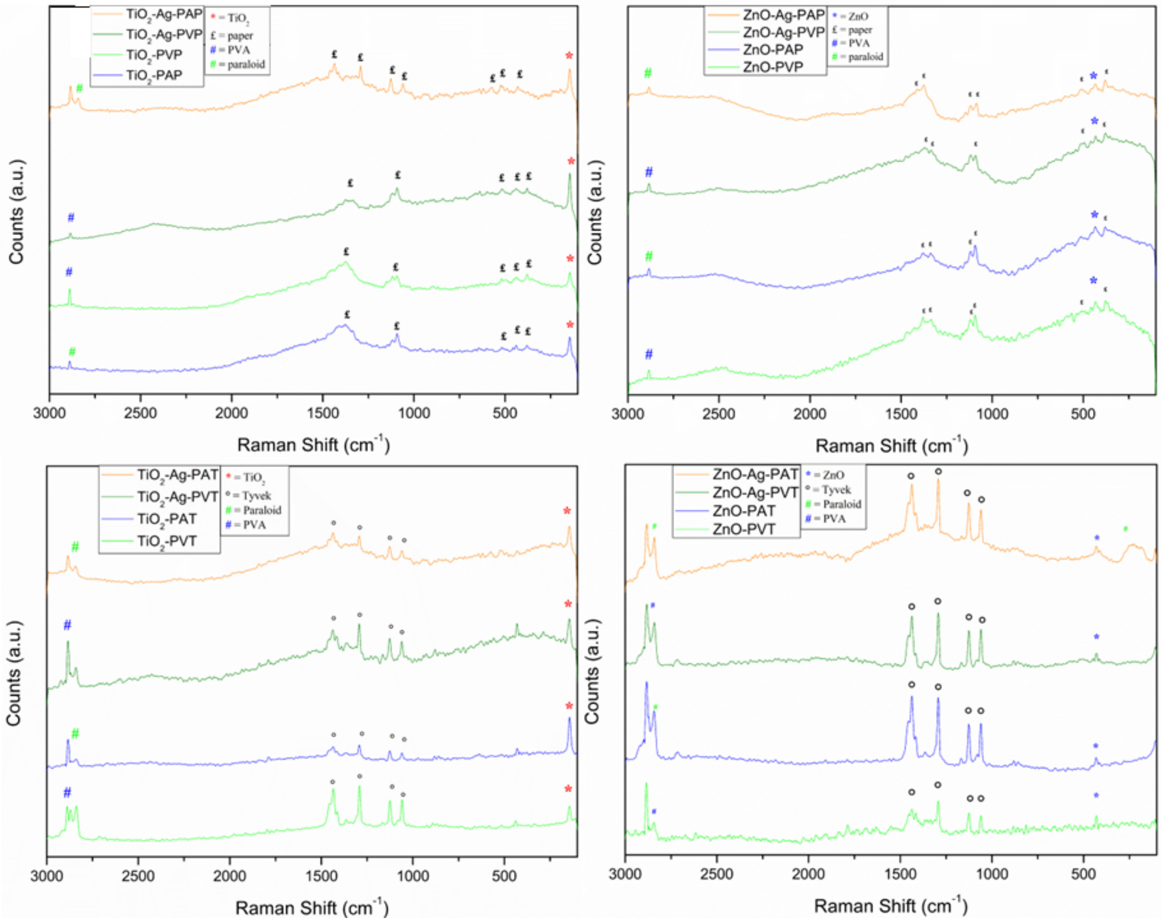

(c) (d)

Figure S1. Micro-Raman spectra: a) paper after TiO_2 NPs and adhesives application; b) paper after ZnO NPs and adhesives application; c) Tyvek® after TiO_2 NPs and adhesives application; d) Tyvek® after ZnO NPs and adhesives application; were PAP is refer to Paraloid on paper, PAT is Paraloid on Tyvek®, PVP is PVA on paper and PVT is PVA on Tyvek®.

Table S1 - Raman peaks of packaging material studied

Sample	Raman shift cm^{-1}	bond	reference
TiO_2-PVP	144	TiO_2	[1], [2]
	1380; 1140; 1074; 520; 443; 380	paper	[3]<br>[4]
	2886	PVA	[5], [6], [7]
TiO_2-Ag-PVP	144	TiO_2	
	1355; 1093; 520; 446; 380	paper	
	2886	PVA	
TiO_2-PAP	144	TiO_2	
	1380; 1074; 520; 443; 383	paper	[3]
	2892	Paraloid B72	[8]
TiO_2-Ag-PAP	144	TiO_2	
	1435; 1296; 1140; 1074; 577; 520; 390	paper	[4]
	2892	Paraloid B72	
ZnO-PVP	437	ZnO	[9]
	1380; 1340; 1130; 1100; 520; 380	paper	
	2886	PVA	
ZnO-Ag-PVP	437	ZnO	
	1380; 1130; 1100; 520; 383	paper	
	2886	PVA	
ZnO-PAP	437	ZnO	
	1380; 1340; 1130; 1100; 520; 443; 380	paper	
	2892	Paraloid B72	
ZnO-Ag-PAP	437	ZnO	
	1415; 1380; 1130; 1100; 520; 383	paper	
	2892	Paraloid B72	
TiO_2-PVT	144	TiO_2	
	1440; 1300; 1130; 1059	Tyvek	[10]
	2886	PVA	
TiO_2-Ag-PVT	144	TiO_2	
	1440; 1300; 1130; 1059	Tyvek	
	2886	PVA	
TiO_2-PAT	144	TiO_2	
	1440; 1300; 1130; 1059	Tyvek	
	2892	Paraloid B72	
TiO_2-Ag-PAT	144	TiO_2	
	1440; 1300; 1130; 1059	Tyvek	
	2892	Paraloid B72	
ZnO-PVT	437	ZnO	
	1440; 1300; 1130; 1059	Tyvek	
	2886	PVA	
ZnO-Ag-PVT	437	ZnO	
	1440; 1300; 1130; 1059	Tyvek	
	2886	PVA	
ZnO-PAT	437	ZnO	
		Tyvek	
	2892	Paraloid B72	
ZnO-Ag-PAT	437	ZnO	
		Tyvek	
	2892	Paraloid B72	

[1] S. Schipporeit and D. Mergel, “Spectral decomposition of <scp>R</scp> aman spectra of mixed-phase <scp> TiO_2 </scp> thin films on <scp>Si</scp> and silicate substrates,” *Journal of Raman Spectroscopy*, vol. 49, no. 7, pp. 1217–1229, Jul. 2018, doi: 10.1002/jrs.5369.

[2] I. Ibarrondo *et al.*, “Portable Raman can be the new hammer for architects restoring 20th-century built heritage elements made of reinforced concrete,” *Journal of Raman Spectroscopy*, vol. 52, no. 1, pp. 109–122, Jan. 2021, doi: 10.1002/jrs.5959.

[3] E. Pigorsch, “New insights into paper—Chemical paper analysis using Raman microscopy,” *Journal of Raman Spectroscopy*, vol. 52, no. 1, pp. 78–84, Jan. 2021, doi: 10.1002/jrs.5877.

[4] B. Nie, Q. Zhou, J. He, and F. Yang, “Chromatographic paper embedded with silver nanostructure as a disposable substrate for surface-enhanced Raman spectroscopy and catalytic reactor,” *Journal of Raman Spectroscopy*, vol. 46, no. 2, pp. 211–216, Feb. 2015, doi: 10.1002/jrs.4636.

[5] S. B. Dangi *et al.*, “Exploration of spectroscopic, surface morphological, structural, electrical, optical and mechanical properties of biocompatible PVA-GO PNCs,” *Diam Relat Mater*, vol. 127, p. 109158, Aug. 2022, doi: 10.1016/j.diamond.2022.109158.

[6] J. Yu, H. Tian, M. Huang, and X. Xu, “Facile Synthesis of Ag NP Films via Evaporation-Induced Self-Assembly and the BA-Sensing Properties,” *Foods*, vol. 12, no. 6, p. 1285, Mar. 2023, doi: 10.3390/foods12061285.

[7] W. I. Singh, S. Sinha, N. A. Devi, S. Nongthombam, S. Laha, and B. P. Swain, “Investigation of chemical bonding and electronic network of rGO/PANI/PVA electrospun nanofiber,” *Polymer Bulletin*, vol. 78, no. 11, pp. 6613–6629, Nov. 2021, doi: 10.1007/s00289-020-03442-7.

[8] X. Bai and V. Detalle, “Time-Gated Pulsed Raman Spectroscopy with NS Laser for Cultural Heritage,” *Heritage*, vol. 6, no. 2, pp. 1531–1540, Feb. 2023, doi: 10.3390/heritage6020082.

[9] C.-Q. Luo, L.-P. Ho, and F. Chi-Chung Ling, “The origin of additional modes in Raman spectra of ZnO:Sb films,” *Physica B Condens Matter*, vol. 593, p. 412256, Sep. 2020, doi: 10.1016/j.physb.2020.412256.

[10] S. Phan, J. L. Padilla-Gamiño, and C. K. Luscombe, “The effect of weathering environments on microplastic chemical identification with Raman and IR spectroscopy: Part I. polyethylene and polypropylene,” *Polym Test*, vol. 116, p. 107752, Dec. 2022, doi: 10.1016/j.polymertesting.2022.107752.
